# The Role of the Trabecular Bone Score in the Assessment of Osteoarticular Disorders in Patients with *HFE*-Hemochromatosis: A Single-Center Study from Poland

**DOI:** 10.3390/genes12091304

**Published:** 2021-08-25

**Authors:** Katarzyna Banaszkiewicz, Katarzyna Sikorska, Damian Panas, Krzysztof Sworczak

**Affiliations:** 1 Department of Tropical Medicine and Epidemiology, Chair of Tropical Medicine and Parasitology, Institute of Martime and Tropical Medicine Gdynia, Faculty of Health Sciences, Medical University of Gdansk, 80-210 Gdansk, Poland; katarzyna.banaszkiewicz@gumed.edu.pl; 2 Department of Tropical and Parasitic Diseases, Chair of Tropical Medicine and Parasitology, Institute of Martime and Tropical Medicine Gdynia, Faculty of Health Sciences, Medical University of Gdansk, 80-210 Gdansk, Poland; 3 Department of Radiological Informatics and Statistics, Medical University of Gdansk, 80-210 Gdansk, Poland; damian.panas@gumed.edu.pl; 4 Department of Endocrinology and Internal Medicine, Medical University of Gdansk, 80-210 Gdansk, Poland; krzysztof.sworczak@gumed.edu.pl

**Keywords:** *HFE* gene, hereditary hemochromatosis, osteoporosis, bone mineral density, trabecular bone score, vitamin D

## Abstract

Type 1 hereditary hemochromatosis (HH) is an autosomal, recessive genetic entity with systemic iron overload. Iron homeostasis disorders develop as a result of *HFE* gene mutations, which are associated with hepcidin arthropathy or osteoporosis and may cause permanent disability in HH patients despite a properly conducted treatment with phlebotomies. In this study, selected parameters of calcium and phosphate metabolism were analyzed in combination with the assessment of bone mineral density (BMD) disorders in patients from northern Poland with clinically overt *HFE*-HH. BMD was determined by a dual-energy X-ray absorptiometry (DXA) test with the use of the trabecular bone score (TBS) function. The study included 29 HH patients (mean age = 53.14 years) who were compared with 20 healthy volunteers. A significantly lower TBS parameter and serum 25-OH-D3 concentration, a higher concentration of intact parathormone and more a frequent occurrence of joint pain were found in HH patients compared with the control group. In HH patients, the diagnosis of liver cirrhosis was associated with lower serum 25-OH-D3 and osteocalcin concentrations. In HH, DXA with the TBS option is a valuable tool in the early assessment of the bone microarchitecture and fracture risk. A supplementation of vitamin D, monitoring its concentration, should be considered especially in HH patients with liver damage and liver cirrhosis.

## 1. Introduction

Hereditary hemochromatosis (HH) develops as a result of genetically determined disorders of iron management. In the classic form of HH (hereditary hemochromatosis type 1; *HFE*-hemochromatosis), the pathology is caused by a genetic defect in the *HFE* gene, inherited autosomal recessively. Most often, HH develops in homozygous carriers of the *HFE* C282Y gene mutation in which, as a result of a point nucleotide change (845G > A), tyrosine is substituted for cysteine in the HFE protein chain, which leads to the loss of its function and consequently to hepcidin deficiency. The prevalence of this mutation in Caucasians is estimated to be 3 to 10 people per 1000, which makes HH one of the first places in terms of the incidence of genetically determined metabolic diseases [1].

The disease may manifest itself with symptoms of liver damage, which in primary untreated HH may lead to liver cirrhosis with the risk of the development of a hepatocellular carcinoma (HCC) [2,3]. The consequences of the progressive iron deposition in other organs are diabetes mellitus, cardiomyopathy, hypopituitarism and other endocrine disorders (primary hypothyroidism and adrenal gland insufficiency, hypogonadism, infertility) and an easily noticeable dark (brown) skin color as well as a pathology of the osteoarticular system, osteopenia and osteoporosis [3]. An early diagnosis and effective treatment protect patients against the development of irreversible organ complications. Joint pain is a frequently reported ailment; it may affect up to 75% of patients even in the period before diagnosis is established. Overt clinical symptoms usually appear in the fifth decade of life although they may appear even in the third decade. Unfortunately, complications related to the musculoskeletal system of *HFE*-hemochromatosis, described mainly in the group of homozygous carriers of the C282Y mutation in the form of arthropathy or osteoporosis, may cause permanent disability despite properly conducted treatment [4,5,6,7,8]. Osteoporosis has been defined by the World Health Organization (WHO) as a progressive systemic disease affecting the skeletal system, characterized by a low bone mass and disturbed microarchitecture, resulting in increased bone fragility and a susceptibility to fractures [9]. The most common definition of osteoporosis used in everyday medical practice is based on the results of a dual-energy X-ray absorptiometry (DXA) examination carried out at the proximal end of the femur or the lumbar spine. The DXA test provides information about bone mineral density (BMD), an important but not the only derivative of bone strength. BMD is determined using the T-score, which is calculated from the number of standard deviations (SD) and the proximity to the average BMD of a young woman aged 20–29. If it is 2.5 SD lower than the norm, osteoporosis is diagnosed and if it is 1.5 SD, osteopenia is diagnosed. This criterion applies to postmenopausal women and men >50 years of age whereas the Z-score is used in younger patients and children [9]. In practice, apart from the DXA test result, there must be additional risk factors for the diagnosis of osteoporosis and this is when secondary osteoporosis is most often diagnosed. However, the failure to meet the densitometric criterion does not exclude the risk of a low-energy fracture. This fracture is the most important clinical symptom of osteoporosis. Osteoporosis should also be diagnosed in people with osteopenia and a low-energy fracture in such locations as the proximal end of the humerus, vertebra, pelvis and distal radius as well as people without fractures but with a high Fracture Risk Assessment Tool (FRAX score). FRAX is the best known method of a fracture risk assessment [10,11].

The most recently introduced tool is the trabecular bone score (TBS) obtained from the spatial grayscale analysis of DXA images. As it enables the evaluation of the bone microarchitecture, the TBS can be useful as an independent and supplementary tool for bone evaluation [12]. A low TBS indicates a weak bone prone to fractures. A low TBS has been shown to be associated with a smaller, more widely distributed and poorly connected trabecula whereas high TBS values correlate with a stronger trabecular structure. Due to the fact that the TBS is a new tool in the diagnosis of osteoporosis, standards are not available for all age groups. For postmenopausal women, it has been established that TBS values > 1.35 are normal, a TBS between 1.2 and 1.35 is associated with a partially disturbed architecture and values below 1.2 definitely indicate a disturbed bone [13,14]. Moreover, according to the available literature, hemochromatosis is associated with an increased risk of joint implantation surgery due to advanced degeneration [8]. The aim of the study was to assess bone mineral density disorders and the TBS as well as the parameters of calcium-phosphate metabolism in relation to the occurrence of joint pain in a group of Polish patients who were residents of the Pomeranian region (northern Poland) diagnosed with *HFE*-hemochromatosis.

## 2. Materials and Methods

Patients over 18 years old diagnosed with *HFE*-hemochromatosis who were under the care of the Hepatology Clinic and treated with bloodlettings were recruited to the study. A total of 29 patients (12 women and 17 men) aged 25–73 years (mean 53, 14 years, median 55) were included; 17 of 40 patients were at an age under 50 years old. The disease was diagnosed based on the presence of elevated serum iron metabolism parameters in blood tests: iron and/or ferritin concentrations in women > 200 ng/mL, in men > 300 ng/mL or transferrin saturation > 45% and the detection of the C282Y/C282Y *HFE* gene mutation. In the study group, an excessive iron accumulation in hepatocytes was confirmed by a histopathological examination of the liver biopsy specimen determined by a semi-quantitative method as previously described [15].

In four patients, the diagnosis was established according to the European Association for the Study of the Liver (EASL) criteria based on the biochemical markers of an increased iron accumulation and genetic testing [16]. Clinical data were obtained from the available, retrospectively analyzed medical records collected at the Hepatology Clinic in Gdansk. As indicators of liver damage, the activity of alanine and aspartate aminotransferase and the results of the assessment of inflammatory activity and fibrosis in the liver biopsy specimen at diagnosis were also taken into account. Liver cirrhosis was diagnosed in five patients on the basis of a histopathological examination. The control group included 20 healthy volunteers aged over 18 years whose sex and age matched the study group with no abnormalities in the laboratory parameters assessing the iron management.

Parameters of calcium-phosphate metabolism included:

- Serum calcium (Ca) concentrations, determined by spectrophotometry; the reference standard was 8.9–10.00 mg/dL.
- Serum phosphorus (Pi) concentration, determined by spectrophotometry; the reference standard was 2.3–4.7 mg/dL;
- Serum osteocalcin concentration, determined by the immunochemiluminescence method (ChLIA); the norms were 3.1–13.7 ng/mL;
- Serum vitamin 25-OH-D3 concentration, determined by the ChLIA method; the norms were 30–80 ng/mL;
- Serum intact parathyroid hormone (PTH int) concentration, determined by the ChLIA method; the norms were 4.6–58.1 pg/mL;
- Alkaline phosphatase (FALK) activity, determined by spectrophotometry; the norms were 44–115 U/L;
- Calcium excretion with diurnal urine (uCa), determined by spectrophotometry; the norms were 100–300 mg/24 h.

To assess BMD, a DXA test with a Hologic Discovery Wi device was used in three locations (femoral neck, distal epiphysis of the humerus, lumbar spine) in the study and control groups at the Densitometry laboratory of the Endocrinology and Internal Diseases Clinic of the University Central Hospital. For the complete diagnosis of osteoporosis, automatic DXA morphometry was used simultaneously to determine BMD in the axial skeleton (VFA: vertebral fracture assessment). This method allows the automatic identification of vertebral body fractures in the thoraco-lumbar region from Th7 to L4. The TBS parameter was used to assess the quality of the trabecular bone in the spine.

The FRAX calculator was used as a method of fracture risk assessment, which estimates the risk of fractures in the next 10 years of a patient.

The T-score and Z-score values were based on the NHANES III (The Third National Health and Nutrition Examination Survey) standard for the female population.

The FRAX scale takes into account:

1. DXA result of the femur;
2. Age;
3. Gender;
4. Body weight;
5. Growth;
6. Clinical risk factors for fractures:
  - Previous fracture;
  - Broken hip in family history (parents);
  - Coexisting diseases, including chronic liver disease;
  - Use of glucocorticoids;
  - Smoking;
  - Alcohol consumption.

The FRAX calculator validated for the Polish population was filled out on the website www.sheffield.ac.uk/FRAX/tool.aspx, accessed on 21 August 2016.

To assess the clinical symptoms of the osteoarticular disorders in HH patients, a questionnaire was conducted containing questions relating to joint pain (hand joints, knees, hip joints).

The following criteria were excluded from the study: patient’s lack of consent to the study, age under 18 and contraindications to the DXA study including pregnancy. The participants gave their written consent to participate in the project. The study was approved by the Independent Bioethical Committee for Scientific Research at the Medical University of Gdansk (NKBBN/117/2016).

### Statistical Methods

The quantitative variables of the independent groups were compared using the Welch test. In the case of unsatisfied assumptions of the Welch test, the non-parametric Mann–Whitney test was used. The normality of the distributions was assessed using the Shapiro–Wilk test and a quantile–quantile plot. The statistically significant results were illustrated with box plots and additional statistics in the form of the effect size (Hedges g or rank-biserial correlation) and a 95% confidence interval of the difference between the means. The relationship between the quantitative variables was determined using Pearson’s linear correlation coefficients. The statistically significant relationships were presented in the scatter plots, which also included the linear regression function along with the 95% confidence interval. The distributions of the dichotomous variables were compared using the chi-squared test and Cramer’s V coefficient. If the assumptions about the minimum number of observations were not met, the Fisher exact test was used instead of the chi-squared test.

All graphs and calculations were made in R 4.1.0, with particular emphasis on the ggstatsplot package [17].

## 3. Results

### 3.1. Description of the Study Group

The mean age at diagnosis was 45.10 years (SD = 12.75) and the time from the first symptoms to the diagnosis of HH was 21.03 months (SD = 17.45). There was no significant difference in the mean age in the study (53.14 years) and control groups (48.1 years) at study enrollment (*p* > 0.05, Welch test). The mean time from the diagnosis of hemochromatosis to the recruitment was 7.07 years (SD = 6.41) and the mean number of phlebotomies was 25. The mean maximum concentration of ferritin was 1014 ng/mL (SD = 901.30) whereas during the treatment with phlebotomies the mean calculated from all measurements performed before the phlebotomies was 601.70 ng/mL (SD = 571.17). A more detailed description of the study group is presented in Table 1.

### 3.2. Osteoporosis and Parameters of Calcium and Phosphate Metabolism

**Figure 1:**
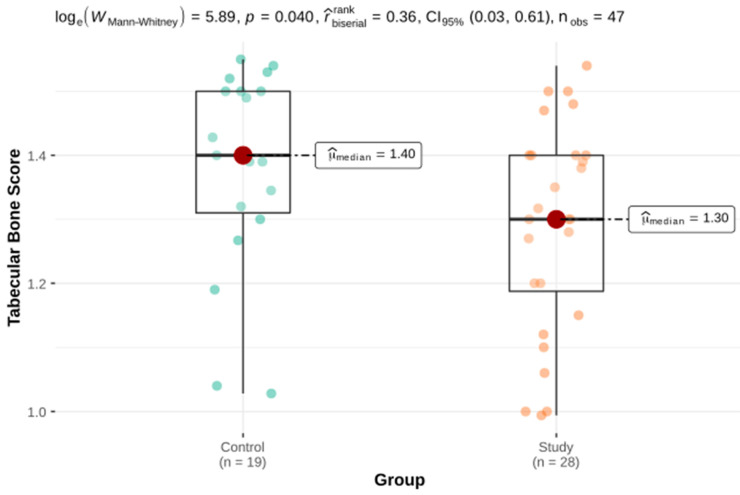
The TBS in the study and the control groups. TBS: trabecular bone score.

**Figure 2:**
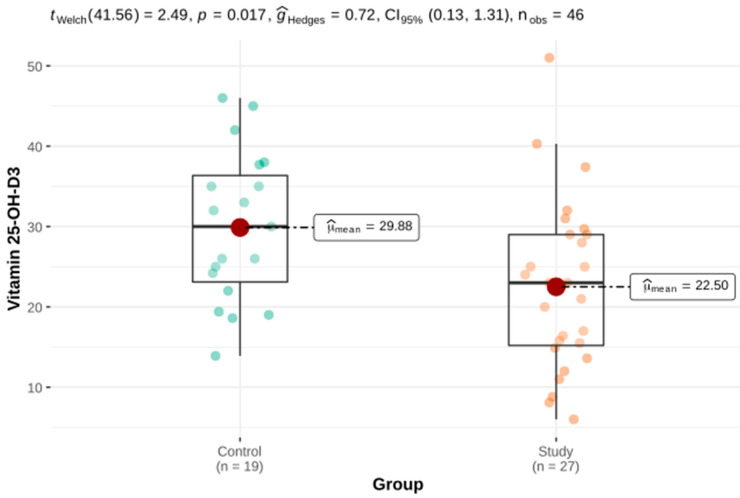
The serum vitamin 25-OH-D3 concentration in the study and the control groups.

**Figure 3:**
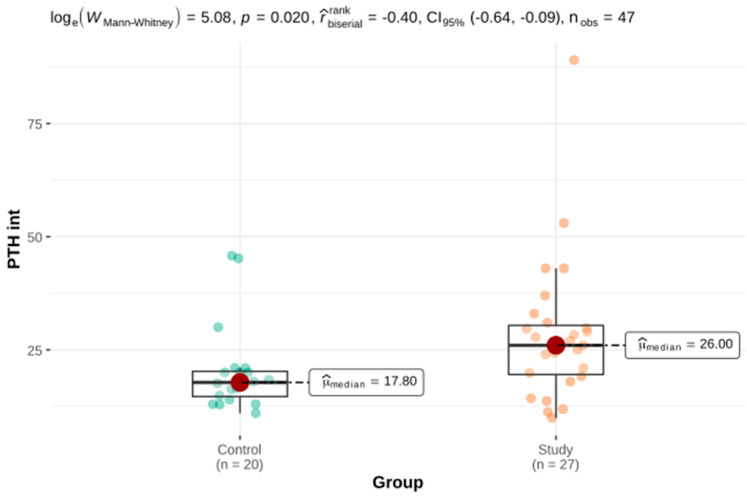
The serum intact parathyroid hormone (PTH int) concentration in the study and the control groups.

Welch’s *t*-test and the Mann–Whitney U test for independent groups were used as part of the comparative analysis of the parameters assessing BMD parameters in three projections as well as the laboratory parameters of calcium-phosphate metabolism. There were statistically significant differences in the TBS, PTH int and serum levels of vitamin 25-OH-D3 between the study group and the control group. There was no significant difference in BMD or FRAX. These results are presented in Table 2 and Figure 1, Figure 2 and Figure 3.

In order to analyze the association of iron overload with osteoporosis and osteopenia, patients with HH were divided into two groups: (1) with diagnosed osteoporosis and osteopenia and (2) the group without these diagnoses. The two groups were compared in terms of the following variables: transferrin saturation, iron concentration, the maximum concentration of ferritin at the diagnosis of hemochromatosis and the concentration of ferritin during bloodlettings and parameters of calcium and phosphate metabolism. There were no statistically significant differences in iron parameters (*p* > 0.05, Mann–Whitney test) between the groups of HH patients (Table 3).

To analyze the association of iron parameters in patients with HH with the parameters of BMD and the laboratory indices of calcium-phosphate metabolism and liver function tests, the correlation coefficients were calculated. We found a significant negative correlation for the T-spine score and transferrin saturation and for 25-OH-D3 and activity of aminotransferases and ferritin concentrations. All results are shown in Table S1.

In the group of HH patients, the diagnosis of osteoporosis based on BMD criteria was not dependent on the intensity of liver iron in the histopathological examination (Figure S1).

**Figure 4:**
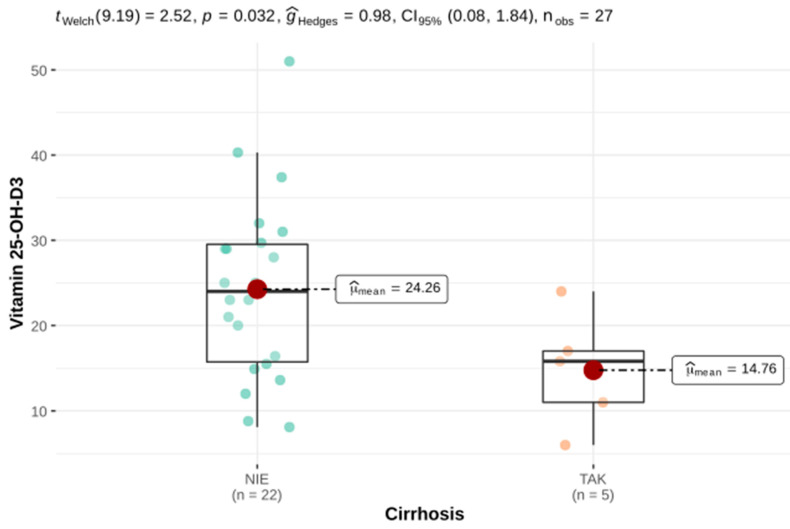
Serum 25-OH-D3 concentration in the group of patients with HH: on the left, patients without liver cirrhosis; on the right, with diagnosed cirrhosis.

**Figure 5:**
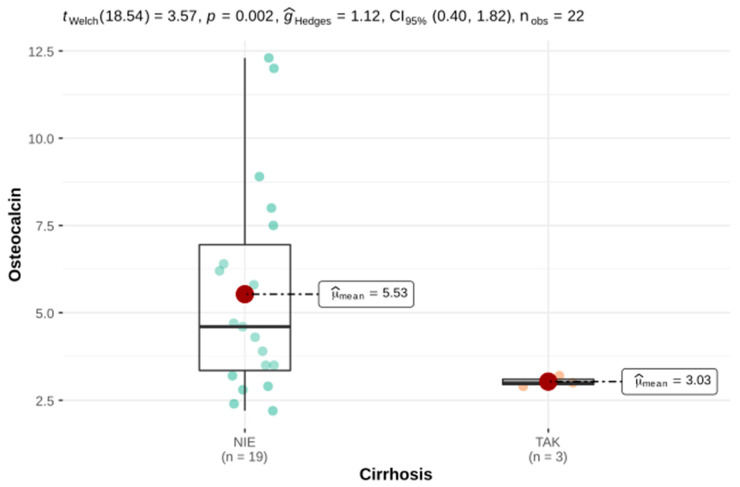
Osteocalcin concentration in the group of patients with HH: on the left, patients without liver cirrhosis; on the right, with diagnosed liver cirrhosis.

Statistically significant lower serum 25-OH-D3 and osteocalcin concentrations were demonstrated in HH patients who were diagnosed with liver cirrhosis compared with HH patients without liver cirrhosis (Figure 4 and Figure 5).

### 3.3. Joint Pain

**Figure 6:**
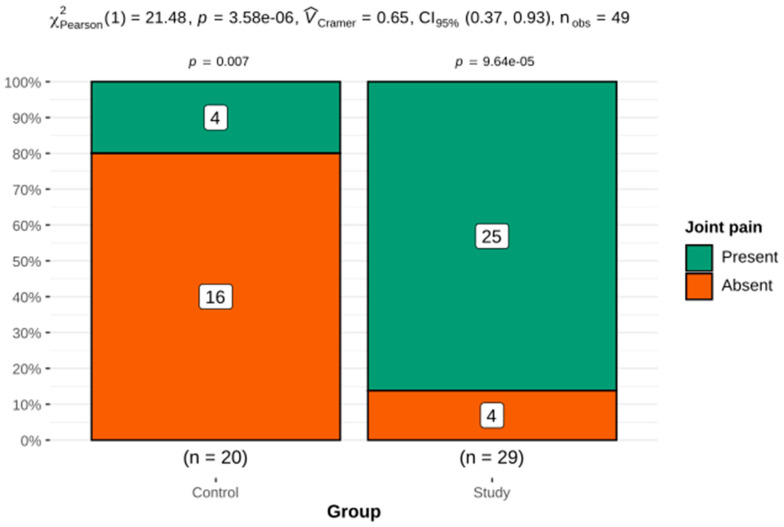
The frequency of joint pain in the HH group compared with the control group.

Joint pain in the group of patients with HH was significantly more frequent than in the control group (Figure 6).

In order to analyze the association of excess iron with the symptoms of the osteoarticular system, patients were divided into groups reporting joint pain and those without such ailments. The parameters of iron and calcium-phosphate metabolism were then assessed in these groups. Excluding the association of joint pain with AST activity, no statistically significant differences in the assessed parameters were found (Table S2).

## 4. Discussion

The study showed no significant differences in the frequency of diagnosis of osteopenia and osteoporosis based on the BMD densitometry criterion between the study and the control groups. There was also no difference in FRAX assessing the fracture risk. On the other hand, the differences in the TBS (i.e., the parameter describing the bone microarchitecture) were statistically significant. It should be emphasized that in the study group the mean TBS index was 1.29 (SD = 0.03) whereas in the control group it was 1.38 (SD = 0.16) (*p* = 0.04). The mean TBS value of patients with HH indicated (if the norms for postmenopausal patients were used) a moderate disturbance of the bone architecture. Moreover, it was found in a group with a mean age of 50 years.

Both a deficiency of and excess iron in the body can adversely affect bones by disturbing the balance between bone resorption and bone regeneration. There is growing scientific evidence that iron overload (HH, thalassemia, aplastic anemia treated with large volumes of concentrated red blood cells) is associated with the weakening of bone tissue, which is represented by a lowering bone mass, osteoporosis, osteopenia and a disturbed bone microarchitecture as well as more frequent fractures. The effect of excess iron on bone tissue is twofold: on the one hand, it inhibits normal bone formation through a reduced osteoblast maturation; on the other hand, by stimulating the maturation of the osteoclast precursors. In addition, there is a direct effect on the RANK receptors (receptor activator of nuclear factor κB-) causes increased bone resorption [18].

Hypogonadism can also lead to bone loss in men that is often seen in patients with HH. In the first retrospective study from 2009 conducted on 87 Italian HH patients, 25% were diagnosed with osteoporosis and 41% with osteopenia. Moreover, it was found that BMD was dependent on a low BMI, the activity of alkaline phosphatase, hypogonadism/menopause and the intensity of the accumulation of iron deposits in the liver. It was not related to a diagnosis of liver cirrhosis, diabetes or cardiomyopathy. It was then concluded that iron overload influenced the etiology of osteoporosis in this group of patients [19].

Advanced densitometric changes in patients with HH were demonstrated by Guggenbuhl et al.: osteopenia was found in 30/38 (78.9%) and osteoporosis in 13/38 (34%) of the patients. The decrease in BMD was more pronounced in the femoral neck than in the lumbar spine. Interestingly, no decreased 25-OH-D3 concentration or abnormal PTH concentration was found in the study group. Bone remodeling was impaired in patients with liver cirrhosis. The authors concluded that HH appeared to be associated with a significant decrease in BMD [20].

The largest clinical trial (600 participants), which focused on the analysis of osteoporosis in patients with HH, confirmed the increased incidence of osteoporosis in HH patients compared with the control group. A disadvantage of this study was that it was based only on a questionnaire filled in by patients regarding symptoms such as osteoporosis and fractures in the hip, wrist and spine. This type of analysis could be burdened with an error related to the insufficient knowledge that the patients had about their own health. In addition, the BMD values were not compared in this study [7].

The analysis of the calcium and phosphate metabolism disorders of HH cannot omit typical coexisting liver damage. For example, in chronic liver disease, 20–100% of patients develop hepatic osteodystrophy, which is caused by disorders of the hepatic hydroxylation of vitamin D, changes in the vitamin D receptor, an increase in serum PTH concentration (as in our study group), IGF-1 deficiency, hyperbilirubinemia, hypogonadism, osteoproteger deficiency, pharmacotherapy and malabsorption [21].

The impact of iron overload on bones has been studied in cell lines and animal models. It has been shown that iron overload suppresses the differentiation and function of the osteoblastic lineage cells, which is related to their more intense apoptosis. Iron also hurts bones through the BMP2 protein (bone morphogenetic protein 2) and osteoblastogenesis. Excessive iron storage may contribute to the development of osteoporosis by inhibiting the proliferation and differentiation of osteoblasts [22,23]. In a study carried out on rats with an *HFE* gene mutation and excessive iron deposition in the kidneys and liver, a reduction in the connections between cells in the trabecular bone within the femur was shown [24].

Doyard et al. used histomorphometry to assess the activity of osteoblasts in mice with the *HFE* -/- phenotype. It was shown that animals with an impaired iron metabolism had a reduced bone formation rate, suggesting a direct influence of iron on the percentage of active osteoblasts. Furthermore, based on a bone mass analysis, it was shown that the bones in animals presenting with an iron overload syndrome has a disturbed microarchitecture due to an impaired trabecular bone formation and a reduced trabecular bone number [25].

The results obtained in our study supported the association of an HH diagnosis with skeletal disorders and they corresponded with the results of other recently published studies. A retrospective analysis of 10 German HH patients from the Bone Disease Clinic showed normal parameters of calcium-phosphate metabolism and markers of bone turnover in most patients. The thickness of the cortical bone, which was assessed using high-resolution peripheral quantitative computed tomography (HRCT), was significantly lower in patients with HH than in the control group, which confirmed the disturbances of the bone microarchitecture in these patients [26].

According to the Manitoba Study, the assessment of the spine TBS predicts fractures almost as well as the BMD test and the use of the TBS and BMD in parallel exceeds the effectiveness of fracture prediction of BMD alone [13]. Therefore, in the algorithm for the management of patients with HH, it would be appropriate for the densitometry test recommended by EASL to pay attention to whether it is possible to use the TBS function in a given device [16].

In our study, the markers of bone turnover (serum osteocalcin, serum FALK and urinary calcium excretion and serum PTH, serum calcium and phosphate) did not differ significantly between the compared groups. However, a significantly lower concentration of osteocalcin was found in the group of patients with HH and liver cirrhosis. Therefore, we can conclude that advanced liver disease, probably due to a reduced secretion of growth factors, exacerbates an adverse inhibitory effect of the iron overload-related inhibition of the bone formation. However, we cannot demonstrate a direct relationship between iron alone and osteoporosis as there were no differences in the diagnosis of osteoporosis in the group of HH patients with a solid and weak liver iron accumulation. However, we adopted the worldwide BMD-based criteria for osteoporosis published by the WHO and other parameters describing bone quality were not considered in this definition of osteoporosis.

As in a Canadian study from 1985, we showed a significantly lower serum 25-OH-D3 concentration in the HH group compared with the control group [27]. HH patients in our study presented higher serum PTH, which could be explained by the state of vitamin D deficiency and, consequently, the synthesis of more parathyroid hormones by the parathyroid glands to maintain a stable serum calcium concentration. In the Polish population, vitamin D deficiency occurs in 90% of the society and in the conditions of chronic liver disease, apart from the population risk, there is an influence of the reduced 25-hydroxylation of vitamin D3. As both chronic liver illness and vitamin D deficiency are risk factors for osteoporosis, it is reasonable to determine and monitor the concentration of 25-OH-D3 in a group of patients with HH and to compensate for deficiencies [28].

Arthropathy and joint pain are among the most severe complaints reported by patients with HH. Joint pain was often reported in our HH group. We did not find a relationship between joint pain and iron parameters. Undoubtedly, joint pain is influenced by the formation of degenerative changes in the form of osteophytes or subchondral cysts, which can cause very severe ailments. Osteomalacia is a syndrome of disturbances in the mineralization of a newly formed osteoid, which may occur in conditions of vitamin D3 deficiency. Apart from bone deformities and fractures, the symptoms of osteomalacia include bone pain, muscle weakness and back pain. A linear relationship was also shown between vitamin D3 deficiency and an impaired liver function. These results are consistent with the current state of knowledge where the role of the liver in the 25-hydroxylation of vitamin D3 is emphasized. Disturbances in this process may be one of the components of so-called hepatic osteodystrophy. In HH patients with liver cirrhosis, low levels of osteocalcin were also present, which is indicative of a reduced bone turnover and may be associated with a poorer quality of bone tissue. This may be related to the lower 25-OH-D3 concentration, as proven in this study, which, by supporting calcium absorption in the intestine, affects bone mineralization.

## 5. Conclusions

There were no significant differences between BMD in the study and the control group in our study, which was in contrast to previously published results obtained in HH patients. Possibly, it is related to the diagnosis of HH among relatively young patients in our study group. On the other hand, a significantly lower TBS in relation to the sex and age-matched control group was demonstrated, which indicated disturbances in the bone microarchitecture in the group of HH patients. In the therapeutic algorithm of patients with HH, DXA with the TBS option is a valuable tool in the early assessment of the bone microarchitecture and fracture risk. Based on the reduced concentration of osteocalcin in the group of patients with HH and liver cirrhosis, we can conclude that a reduction of bone turnover and the inhibition of bone formation are exacerbated in this subgroup of patients with a genetic iron overload. A supplementation of vitamin D, monitoring its concentration, should be considered especially in HH patients with liver damage and liver cirrhosis.

## Figures and Tables

**Table 1:** Characteristics of the study group.

	Mean	Me	Min–Max
Age at study entry (years)	53.14	55	25–73
Age at diagnosis (years)	45.10	46	23–70
Number of phlebotomies	25	14.5	1–178
Maximum ferritin concentration (women ref. standards 15–200 µg/L; men 150–300 µg/L)	1014	800	126–3550
Average ferritin concentration (μg/L)	601.70	358	60–2183
ALT (0–41 U/L)	40	34	12–117
AST (0–40 U/L)	31.83	28	8–77
Transferrin saturation (20–40%)	86.59	90	60–100

Me: median; min: smallest value; max: largest value; ALT: alanine aminotransferase; AST: aspartate aminotransferase. Numbers in brackets are the units and standards for laboratory determinations.

**Table 2:** Densitometric and laboratory parameters of the calcium and phosphate metabolism in the study and control groups.

	Study Group					Control Group					Difference	
	N	Mean ± SEM	Q1	Me	Q3	N	Mean ± SEM	Q1	Me	Q3	Stat	*p*
BMD femur	29	0.83 ± 0.02	0.73	0.81	0.90	19	0.82 ± 0.02	0.73	0.80	0.95	0.19 ^a^	0.846
BMD lumbar spine	28	1.01 ± 0.03	0.90	1.03	1.13	19	1.05 ± 0.04	0.91	1.08	1.18	0.67 ^a^	0.508
BMD forearm	29	0.73 ± 0.02	0.65	0.74	0.82	19	0,73 ± 0.02	0.64	0.74	0.81	0.13 ^a^	0.897
TBS	28	1.29 ± 0.03	1.19	1.30	1.40	19	1.38 ± 0.03	1.31	1.40	1.50	361 ^b^	0.040
FRAX major (%)	29	4.71 ± 1.23	1.0	3.2	4.7	19	2.42 ± 0.54	0.75	1.4	3.45	200.5 ^b^	0.116
FRAX femur (%)	29	0.61 ± 0.19	0.1	0.3	0.6	19	0.37 ± 0.14	0.0	0.1	0.3	210.5 ^b^	0.166
Ca (mg/dL)	27	9.62 ± 0.07	9.3	9.7	9.9	20	9.79 ± 0.09	9.57	9.79	10.02	336 ^b^	0.156
Pi (mg/dL)	27	3.11 ± 0.09	2.85	3.1	3.4	20	3.1 ± 0.14	2.67	3.05	3.6	0.06 ^a^	0.954
25-OH-D3 (ng/mL)	27	22.5 ± 2.04	15.2	23	29	19	29.88 ± 2.15	23.1	30	36.35	2.49 ^a^	0.017
FALK (U/L)	27	76.04 ± 7.45	50	66	89	20	66.05 ± 3.80	53.25	66	78.25	261.5 ^b^	0.863
Calcium urine excretion (mg/24 h)	24	153.1 ± 14.91	107.5	118.5	185	19	155.2 ± 17.28	103	138	204.5	237 ^b^	0.835
Osteocalcin (ng/mL)	22	5.19 ± 0.63	3.05	4.1	6.35	18	5.71 ± 0.67	3.25	4.9	6.875	229.5 ^b^	0.399
PTH int (pg/mL)	27	28.31 ± 3.05	19.55	26	30.40	20	20.3 ± 2.14	14.68	17.8	20.25	161.5 ^b^	0.020

BMD: bone mineral density; TBS: trabecular bone quality; FRAX: fracture risk calculator; FALK: alkaline phosphatase; PTH int: intact parathyroid hormone; Me: median; Q1: lower quartile; Q3: upper quartile; SEM: standard error of the mean; N: number of patients; Stat: statistic of ^a^ Welch’s *t*-test, ^b^ Mann–Whitney U test. Statistically significant differences between the groups are marked in red (*p* < 0.05).

**Table 3:** Selected liver function tests, iron parameters and calcium-phosphate metabolism in HH patients in the groups with osteoporosis/osteopenia and without osteoporosis/osteopenia.

	Osteoporosis/Osteopenia					No Osteoporosis/Osteopenia					Difference	
	N	Mean ± SEM	Q1	Me	Q3	N	Mean ± SEM	Q1	Me	Q3	U	*p*
GGTP (U/L)	12	32.75 ± 5.78	19.0	32.75	37.75	15	47.67 ± 12.29	23.5	35	49.5	102	0.574
Bilirubin (mg/dL)	12	0.86 ± 0.13	0.6	0.75	0.925	15	0.78 ± 0.05	0.67	0.73	0.8	88	0.941
AST (U/L)	12	27.92 ± 3.35	21.25	24.5	30.75	16	330.06 ± 4.59	19.75	29.5	39	109.5	0.545
ALT (U/L)	12	33.17 ± 5.99	14.75	22	51.75	16	43.81 ± 7.78	20.75	35	56.25	115.5	0.378
Ferritin max (μg/L)	12	1101.6 ± 327.43	402.5	712.5	1065	16	949 ± 187.46	481	725	1094	96.5	0.999
Ferritin average (μg/L)	12	604.2 ± 168.96	247.8	377.5	659.5	16	604.9 ± 149.41	289.8	337.5	568.8	99	0.907
FALK (U/L)	12	90.25 ± 14.12	56.75	76	94	14	62.5 ± 6.44	48	55	68.25	46.5	0.057
Fe (μg/dL)	12	199.7 ± 16.27	194.8	212	224.8	16	235.2 ± 8.88	205	238.5	261.5	131.5	0.104
Transferrin sat. (%)	12	87.67 ± 3.49	85	90.5	95.75	16	85.75 ± 2.99	77.5	90	93.5	85.5	0.641
PTH int (pg/mL)	12	26.81 ± 3.28	17.98	27.5	34	14	30.34 ± 5.19	21.75	26.5	28.82	80	0.857
Ca (mg/dL)	12	9.62 ± 0.12	9.375	9.65	9.925	14	9.62 ± 0.10	9.3	9.75	9.875	81	0.897
Pi (mg/dL)	12	3.02 ± 0.13	2.825	3.05	3.15	14	3.2 ± 0.12	2.85	3.1	3.5	100.5	0.408
25-OH-D3 (ng/mL)	12	24.51 ± 2.72	16.25	25.5	30.02	14	20.67 ± 3.17	12.4	20.5	25.0	60	0.226

Me: median; Q1: lower quartile; Q3: upper quartile; SEM: standard error of the mean; N: number of patients; U: statistics of the Mann–Whitney test; N: number of patients; GGTP: γ-glutamyl-trans-peptidase; AST: aspartate aminotransferase; ALT: alanine aminotransferase; FALK: alkaline phosphatase; Fe: iron; PTH int: intact parathyroid hormone; Ca: serum calcium; Pi: serum phosphates; *p* < 0.05.

## Supplementary Materials

The following are available online at https://www.mdpi.com/article/10.3390/genes12091304/s1, Figure S1: Osteoporosis/osteopenia in relation to iron accumulation in the liver biopsy specimen in HH patients. Table S1: Bone mineral density, TBS and FRAX values in relation to iron parameters and liver function parameters in patients with HH. Table S2: Selected liver function tests, iron parameters and calcium-phosphate metabolism in HH patients in the groups with joint pain present and without joint pain.
