# Supplementary material for: The Role of the Trabecular Bone Score in the Assessment of Osteoarticular Disorders in Patients with HFE-Hemochromatosis: A Single-Center Study from Poland
Source: Genes (Basel). 2021 Aug 25;12(9):1304. doi: 10.3390/genes12091304 (PMC8470067; doi:10.3390/genes12091304)
Supplement: Supplementary file 1 [file genes-12-01304-s001.zip › genes-1331066-supplementary.pdf]

Table S1. BMD, TBS, FRAX, calcium-phosphate metabolism values in relation to iron parameters and liver function tests in patients with HH.

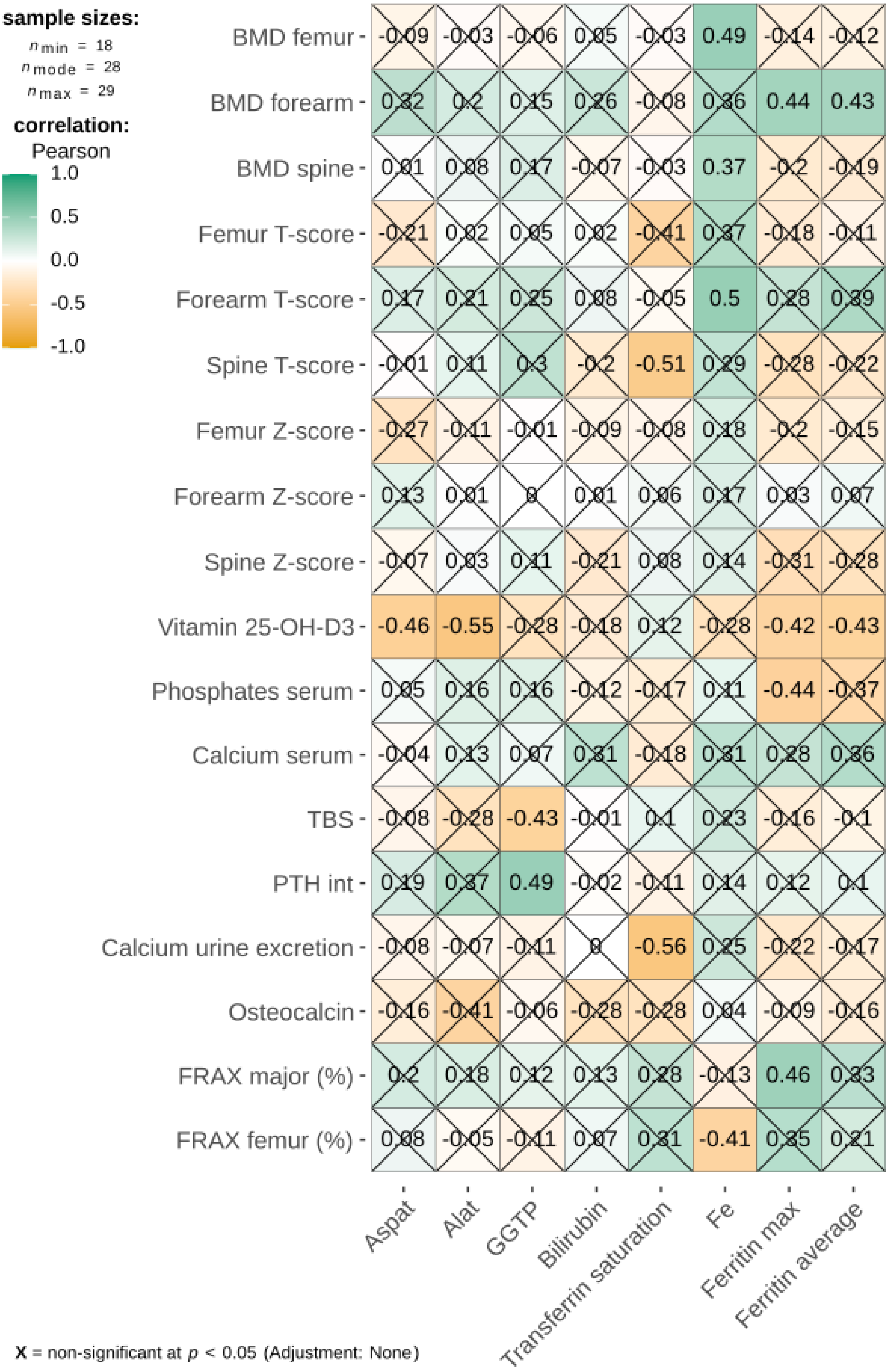

Pearson's correlation coefficients, p-value, and N = number of observations taken to derive a given correlation. Statistically significant results are not crossed, for positive correlation green colour, for negative orange

Table S2. Selected liver function tests, iron parameters and calcium-phosphate metabolism in HH patients in the groups with joint paint present and without joint paint.

	Joint pain present					Joint pain absent					Difference	
	N	Mean ± SEM	Q1	Me	Q3	N	Mean ± SEM	Q1	Me	Q3	U	p
GGTP (U/l)	24	44.71 ± 8.08	19	36	56.75	4	23.5 ± 5.04	16	23.5	31	23	.107
Bilirubin (mg/dl)	24	0.81 ± 0.07	0.60	0.75	1.90	4	0.86 ± 0.14	0.72	0.74	0.89	56	.621
Aspat (U/l)	25	29.56 ± 3.13	19	26	33	4	46.00 ± 7.52	37	43.5	52.5	83	.039
Alat (U/l)	25	39.52 ± 5.69	15	31	54	4	43.00 ± 9.22	31	43.5	55.5	61	.506
Ferrytyna max (µg/l)	25	1083 ± 189.28	500	802	1104	4	582.8 ± 179.69	370	536	748	34	.327
Ferrytyna śr (µg/l)	25	650.8 ± 120.10	260	420	658	4	295 ± 58.93	257.8	307	344.2	32	.268
FALK (U/l)	25	79.04 ± 8.18	51.5	67.5	90	4	52 ± 7.21	45	48	57	15	.113
Fe (µg/dl)	25	216.5 ± 9.83	196	216.5	250	4	234.2 ± 18.71	213.2	240.5	261.5	60	.548
Transferrin saturation (%)	25	88.2 ± 2.04	82	90	95	4	76.5 ± 8.15	63.75	77.5	90.25	26.5	.144
PTH int (pg/ml)	24	28.91 ± 3.37	19.73	25.6	31.5	3	23.5 ± 4.91	20.75	27.80	28.40	32	.787
Ca (mg/dl)	24	9.63 ± 0.08	9.3	9.75	9.9	3	9.70 ± 0.15	9.5	9.7	9.75	31	.727
Pi (mg/dl)	24	3.07 ± 0.09	2.78	3.05	3.3	3	3.43 ± 0.23	3.25	3.5	3.65	51	.259
25-OH-D3 (mg/ml)	24	22.91 ± 2.18	15.72	23	29	3	19.2 ± 6.41	12.8	13.6	22.8	29	.616

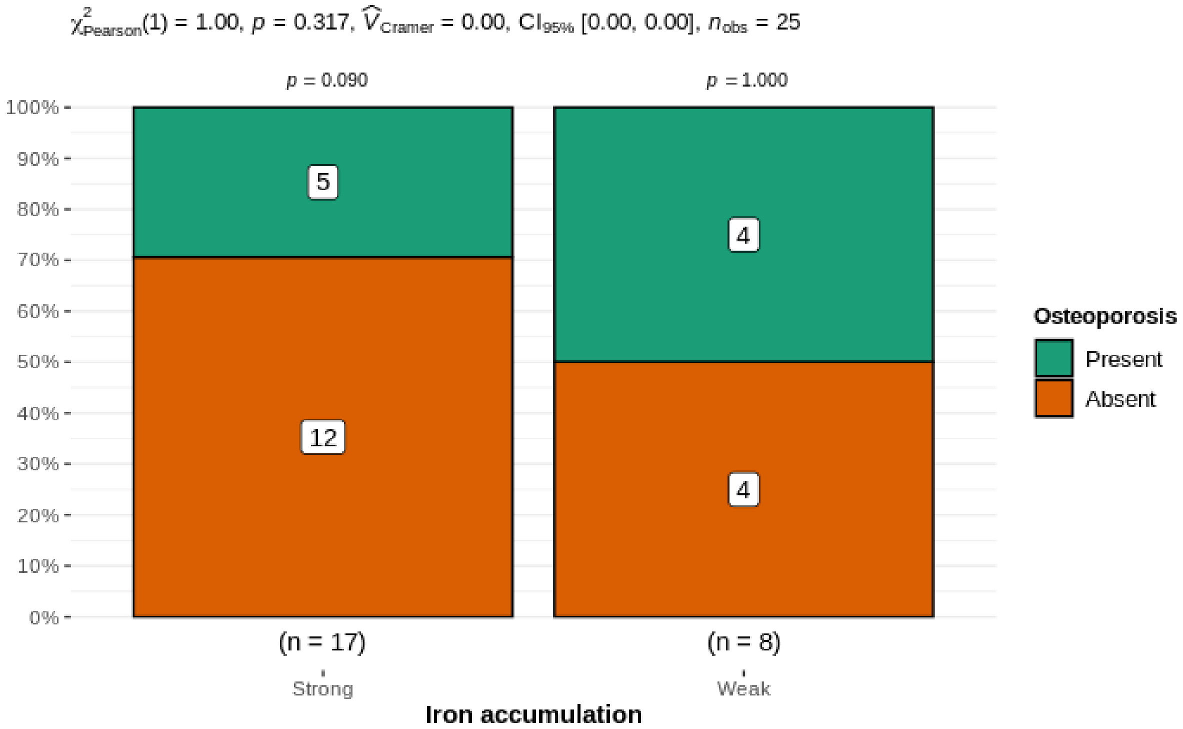

Figure S1. Osteoporosis/osteopenia in relation to iron accumulation in the liver biopsy specimen in HH patients. Left: strong iron accumulation in the liver (G3, G4). Right: weak iron accumulation (G1, G2). (p>0,05, Fisher's exact test).
